# Chemical reversion of age-related oocyte dysfunction fails to enhance embryo development in a bovine model of postovulatory aging

**DOI:** 10.1007/s10815-024-03151-4

**Published:** 2024-06-01

**Authors:** Ana Filipa Ferreira, Juliana Machado-Simões, Inês Moniz, Maria Soares, Alexandra Carvalho, Patrícia Diniz, João Ramalho-Santos, Ana Paula Sousa, Luís Lopes-da-Costa, Teresa Almeida-Santos

**Affiliations:** 1 Reproductive Medicine Unit, Gynecology, Obstetrics, Reproduction and Neonatology Department, Unidade Local de Saúde de Coimbra, Praceta, R. Prof. Mota Pinto, Coimbra, 3004-561 Portugal; 2 Faculty of Medicine, University of Coimbra, Azinhaga de Santa Comba, Celas, Coimbra, 3000-548 Portugal; 3 CNC-UC - Center for Neuroscience and Cell Biology, University of Coimbra, Coimbra, Portugal; 4 CIBB - Centre for Innovative Biomedicine and Biotechnology, University of Coimbra, Coimbra, Portugal; 5 EUGIN Coimbra, Filipe Hodart N° 12, 3000-185 Coimbra, Portugal; 6 Institute for Interdisciplinary Research, Doctoral Programme in Experimental Biology and Biomedicine (PDBEB), University of Coimbra, Coimbra, Portugal; 7 Department of Life Sciences, Calçada Martim de Freitas, University of Coimbra, 3000-456, Coimbra, Portugal; 8 CICS-UBI-Health Sciences Research Centre, University of Beira Interior, Covilhã, Portugal; 9 CIISA – Centre for Interdisciplinary Research in Animal Health, Faculty of Veterinary Medicine, University of Lisbon, Avenida da Universidade Técnica, 1300-477, Lisbon, Portugal; 10 AL4AnimalS – Associate Laboratory for Animal and Veterinary Science, Avenida da Universidade Técnica, 1300-477, Lisbon, Portugal

**Keywords:** Oocyte aging, Oxidative stress, Mitochondrial dysfunction, Antioxidants, Resveratrol, Phloretin

## Abstract

**Purpose:**

There are no clinical treatments to prevent/revert age-related alterations associated with oocyte competence decline in the context of advanced maternal age. Those alterations have been attributed to oxidative stress and mitochondrial dysfunction. Our study aimed to test the hypothesis that in vitro maturation (IVM) medium supplementation with antioxidants (resveratrol or phloretin) may revert age-related oocyte competence decline.

**Methods:**

Bovine immature oocytes were matured in vitro for 23 h (young) and 30 h (aged). Postovulatory aged oocytes (control group) and embryos obtained after fertilization were examined and compared with oocytes supplemented with either 2 μM of resveratrol or 6 μM phloretin (treatment groups) during IVM.

**Results:**

Aged oocytes had a significantly lower mitochondrial mass and proportion of mitochondrial clustered pattern, lower ooplasmic volume, higher ROS, lower sirtuin-1 protein level, and a lower blastocyst rate in comparison to young oocytes, indicating that postovulatory oocytes have a lower quality and developmental competence, thus validating our experimental model. Supplementation of IVM medium with antioxidants prevented the generation of ROS and restored the active mitochondrial mass and pattern characteristic of younger oocytes. Moreover, sirtuin-1 protein levels were also restored but only following incubation with resveratrol. Despite these findings, the blastocyst rate of treatment groups was not significantly different from the control group, indicating that resveratrol and phloretin could not restore the oocyte competence of postovulatory aged oocytes.

**Conclusion:**

Resveratrol and phloretin can both revert the age-related oxidative stress and mitochondrial dysfunction during postovulatory aging but were insufficient to enhance embryo developmental rates under our experimental conditions.

**Supplementary Information:**

The online version contains supplementary material available at 10.1007/s10815-024-03151-4.

## Introduction

Female age is the most important predictor of in vitro fertilization success [1–5] but there are no treatments available to overcome the oocyte competence decline associated with advanced maternal age (AMA). In addition to aneuploidy [6], several age-related alterations have been reported in oocytes from older females, affecting nuclear and cytoplasmic maturation, as well as the oocyte microenvironment. Those alterations have been attributed to different molecular and biological mechanisms, including oxidative stress and mitochondrial dysfunction, as recently reviewed by Ferreira et al. [7].

Oxidative stress affects the oocyte and all ovarian compartments, including granulosa cells (GC), cumulus cells (CC), follicular fluid, and interstitial tissue, as evidenced by protein, lipid, and DNA damage in GC [reviewed by: [8, 9] and ovarian tissue from mouse [10]]. Despite the latest findings that oocytes from early follicles preserve their developmental potential for decades by suppressing complex I of the ETC and thus evading ROS production [11], Smits et al. [12] have recently showed that primordial follicles of AMA women still exhibit the aging phenotype and present markers of protein oxidation and lipid peroxidation. Although these authors failed to demonstrate an age-related increase of DNA oxidation, it has been postulated by others [13] that primordial follicles, which are sensitive to apoptosis in response to DNA, could have undergone apoptosis and not be present in the samples analyzed. In fact, oocytes from older women exhibit mitochondrial DNA mutations [14–17].

Several studies reported higher levels of reactive oxygen species (ROS) [18–21] and compromised antioxidant capacity [22–24]. Importantly, Smits et al. [12] found a decrease in glutathione (GSH) abundance in metaphase I (MI) oocytes of AMA, which is the major nonenzymatic antioxidant found in oocytes [25] suggesting an increase oxidative stress. These authors also reported other age-related alterations in cellular metabolism indicating mitochondrial dysfunction, such as impaired nicotinamide adenine dinucleotide (NAD^+^). NAD^+^ is an important co-enzyme implicated in multiple metabolic processes and a co-factor/substrate for crucial functions in cell signaling pathways, including sirtuins (SIRTs) activity [26].

SIRTs are NAD-dependent deacetylases that sense and respond to alterations of ROS levels, regulating mitochondrial biogenesis and redox homeostasis [27, 28]. Harsh oxidative stress increases SIRT degradation and accumulation of ROS, responsible for damaging biomolecules and organelles. Indeed, lower levels of SIRTs have been reported in aged oocytes [21, 29, 30]. Oxidative stress during oocyte growth disturbs the fundamental mission of providing enough mitochondria for early embryo development and functional mitochondria for ATP supply during oocyte maturation, fertilization, and postfertilization events, hence decreasing oocyte competence [31–34].

Since oxidative stress and mitochondrial dysfunction have been consistently associated with oocyte aging, a growing number of investigations are exploring the use of mitochondrial supplementation (transfer into the oocyte) and antioxidants as potential therapies for oocyte rejuvenation.

The first is founded on increasing the number of healthy/active mitochondria in the oocyte at the time of intracytoplasmic sperm injection (ICSI), along with spermatozoa. However, a well-designed randomized clinical study (RCT) performed in Spain by Labarta et al. (*n* = 57) reported a similar cumulative ongoing pregnancy rate. The pregnancy and live birth rates per transferred embryo were the same, 41.2% in the autologous germline mitochondrial transfer (AUGMENT) group and 41.7% in the control group [35]. More recently, Morimoto et al. evaluated 52 patients and compared the results with previous cycles (before mitochondrial transfer), demonstrating an improvement in clinical pregnancy (from 0 to 27.4%) and live birth rates (from 0 to 21.5%) [36]. Therefore, the efficacy of this procedure as a complementary treatment for assisted reproductive technologies (ART) is still controversial.

Several antioxidants have been tested for the improvement of female fertility in the context of aging, including melatonin, co-enzyme Q10, NAD precursors, N-acetyl-l-cysteine (NAC), quercetin, and resveratrol [37–41]. Antioxidants protect cells from oxidative stress by directly scavenging ROS, thus inhibiting oxidation of biomolecules, and/or by rescuing the activity of antioxidant enzymes. In addition, antioxidants have been shown to increase the expression of SIRTs, promoting mitochondrial biogenesis and function and contributing to ROS homeostasis [37, 42].

However, reproductive outcomes in aged females are still not convincing and require further investigation. Indeed, according to a Cochrane review, there is limited evidence in support of supplemental oral antioxidants for subfertile women [43]. Nonetheless, one of the more discussed antioxidants in this context, resveratrol, was not included in this review.

Resveratrol is a polyphenolic antioxidant and SIRT1 activator found in a variety of plants that has attracted the attention of various researchers for its life-span-extending effects in budding yeast [44]. Resveratrol regulates several synergistic pathways that control metabolism, immune cells, and cell proliferation and apoptosis. Currently, hundreds of clinical trials have been conducted to study its protective effect against numerous diseases, with promising results [45, 46]. Supplementation of oocytes with resveratrol has been shown to reduce ROS levels and improve embryo development [47–50]. In aged oocytes, some authors have also reported an improvement in fertilization and embryo rates [40, 51, 52], whereas others have not evaluated this effect [53] or failed to demonstrate a benefit in oocyte developmental competence [54]. In mice, intraperitoneal injections of resveratrol during a short period have led to decreased ROS levels, although with no differences in reproductive outcomes [55] while oral supplementation for longer periods resulted in a higher number of oocytes and pups [56, 57]. Oral supplementation of AMA women undergoing assisted reproductive technology (ART) treatments with resveratrol resulted in higher fertilization rates, although with similar number of mature oocytes and pregnancy rates in comparison to control group [58]. This is consistent with the results of a recent RCT in which the authors reported no differences in pregnancy rate despite the higher number of oocytes (and MII oocytes) and higher fertilization and blastocyst rates in the resveratrol group. In this study, patients had 18–42 years old and normal ovarian reserve [59]. The biological improvement (i.e., oocyte and embryo quality) was also reported in another RCT [60]. Therefore, there is evidence that resveratrol enhances mitochondria and reduce oxidative stress in the oocyte, but its efficacy in improving oocyte quality remains to be elucidated.

Another polyphenol, phloretin, is produced by apples to protect the plant against environmental insults such as ultraviolet radiation, explaining why phloretin has earned the attention of researchers in the field of dermatology and cosmetic industry (by preventing photoaging) [61]. There are no human studies exploiting the nutraceutical effect of phloretin and no published studies (in vivo or in vitro) in the reproductive field. Regardless, in vivo and in vitro studies have confirmed the direct antioxidant capacity of phloretin by scavenging ROS, as well as the upregulation of antioxidant enzymes [62, 63]. Thus, phloretin can potentially enhance oocyte mitochondrial function by preventing the deleterious effect of oxidative stress on mitochondrial activity, but the effect of this molecule on reproductive cells (e.g., oocyte and *cumulus* cells) is completely unknown.

Our study aimed to test the hypothesis that in vitro maturation (IVM) medium supplementation with antioxidants (resveratrol or phloretin) in a model of postovulatory aging may prevent oxidative stress, and age-related decrease in mitochondrial mass and distribution, sirtuin levels, and embryo developmental rates.

## Materials and methods

This study was approved by the institutional review board of the University of Coimbra, Faculty of Medicine (CE-081/2017).

### Experimental design

Bovine immature cumulus-oocyte complexes (COCs) were gathered, selected, and in vitro matured (as previously described [64]). Matured oocytes were obtained after 23 h of IVM (young oocytes). Extending IVM beyond this time window has been associated with several age-related alterations [64, 65]. Therefore, to mimic oocyte aging, we prolonged IVM for 30 h (aged oocytes). After the maturation period, COCs were denuded, and oocytes from the young versus aged groups were used to evaluate and compare ooplasmic volume, mitochondrial features, and sirtuin expression.

After the establishment of an in vitro model of aging we sought to test the possibility of preventing age-related alterations by supplementation of IVM medium with an antioxidant. Therefore, we matured COCs for 30h in standard IVM medium (aged group, control group) and in IVM medium supplemented with either resveratrol (aged-Res) or phloretin (aged-Ph).

### Reagents

Unless otherwise noted, all chemicals and reagents were obtained from Sigma-Aldrich (Merck, Darmstadt, Germany).

### Bovine ovary collection, COCs aspiration, and selection

Ovaries were obtained from adult cows (*Bos taurus*), with 30–120 months of age, at a local slaughterhouse (Matadouro da Beira Litoral, Aveiro) and transported to the laboratory within 1–2 h. The biological samples were kept in Hanks’ Balanced Salt Solution (HBSS, Gibco-Invitrogen, Thermo Fisher Scientific, Waltham, Massachusetts, USA) supplemented with 0.05 mg/mL kanamycin (K0254) and 0.15% bovine serum albumin (BSA).

Ovaries were washed twice with fresh transport medium warmed to 37°C. COCs were aspirated from 2- to 6-mm antral follicles using a 10-mL syringe with a 19-gauge needle. Thereafter, punctured follicular fluid was diluted in washing medium constituted of HEPES buffered Tissue Culture Medium 199 (TCM 199) supplemented with 10% fetal bovine serum (FBS; S0615, Biochrom, Cambridge, UK), 5 IU/mL heparin, 0.2 mM sodium pyruvate (P2256), 0.04 mg/mL gentamycin (G1272), and 2.5 μg/mL amphotericin B (A2942). Only COCs with homogeneous ooplasm and compact multi-layered *cumulus* were selected.

### IVM and supplementation of IVM medium with resveratrol/phloretin

Groups of 20–25 COCs were placed in individual wells of a 4-well plate containing previously equilibrated IVM medium, kept at 38.5 °C in an atmosphere of 5% CO_2_ and maximum humidity. The IVM medium was composed of TCM199 supplemented with 15% FBS, 50 ng/mL epidermal growth factor (EGF), 10 IU/mL recombinant follicle-stimulating hormone (rFSH;Gonal-F, follitropin-alpha, Merck, Amsterdam, The Netherlands), 5 IU/mL human Choriogonadotropin alfa (hCG;Ovitrelle, Merck), 1 μg/mL β-estradiol, 0.3 mM sodium pyruvate, and 0.04 mg/mL gentamycin (G1272).

Resveratrol was dissolved in ethanol at 110 mM and stored at − 20 °C. Phloretin was dissolved in ethanol at 100 mM and stored at − 20 °C. The optimal resveratrol and phloretin concentrations were determined using dose–response gradients. According to the maturation rates, the proportion of morphologically aberrant oocytes (irregular ooplasm, fragmental cells) and the percentage of ROS-stained oocytes (Supplemental Table 1—resveratrol, Supplemental Table 2—phloretin) we chose the doses of 2 μM for resveratrol and 6 μM for phloretin to proceed with the experiments.

After the IVM period, the oocytes were recovered by repetitive pipetting of the COCs to separate the expanding layers of *cumulus* cells from the oocytes.

### Mitochondrial mass, distribution, and pattern

The oocyte mitochondria were stained with MitoTracker™ Red CMXRos (M7512, Thermo Fisher). Oocytes were incubated with 100 nM of MitoTracker™ Red CMXRos diluted in TCM199 for 40 min at 38.5 °C in maximum humidity. Oocytes were then washed twice in PBS and fixed in 4% paraformaldehyde (PFA) diluted in PBS for 15 min at room temperature. Following two washes, oocytes were mounted on a glass slide in a 10-μL drop of mounting medium Fluoromount, covered with a coverslip and visualized under an epifluorescence microscope (Zeiss, Axio Imager Z2, Munich, Germany). The red fluorescence was observed using a 555/30 (excitation) and 592/25 (emission) filter. The images were captured by the image software AxioVision™ (version 4.8, Zeiss) and the fluorescence intensity was measured using ImageJ™ (v1.53e; NIH, Bethesda, MD, USA).

Mitochondrial mass was quantified in each oocyte by measuring the mean fluorescence intensity (MFI), in arbitrary units, after defining the ooplasm as the region of interest. The groups were compared using *t*-test after setting the MFI in the young group as 1.0.

Oocytes were classified according to their *mitochondrial pattern* as smooth, when a regular mitochondrial signal was identified, or clustered (Fig. 2(A2)), when the mitochondrial signal was characterized by clusters or small clumps. *Mitochondrial distribution* was categorized as homogenous (evenly distributed mitochondria in the ooplasm), non-homogenous (mitochondria lacking in many areas of the ooplasm), or peripheric (large proportion of mitochondria localized in the periphery) (Fig. 2(B2)). The groups were compared using the chi-squared test. When assumptions were not met, data were analyzed in 2 × 2 contingency tables using Fisher’s exact test. This experiment was replicated three times.

### Ooplasmic volume measurement

Oocytes were observed in a drop of PBS under Axio Imager Z2 optical microscope at 400 × magnification. The ooplasm diameters were visualized and the long and minor axis of each oocyte were measured. The ooplasmic volume (μm^3^) of mature oocytes (with visualized first polar body) was evaluated according to Murakoshi et al. [66] using the following formula, in which *D* corresponds to the long axis and *d* is the minor axis:

$$V= \frac{\pi \times {d}^{2} \times D}{6} , \pi =3.14$$

Young and aged groups were compared using* t*-test, as well as aged-Res and aged-Ph groups, that were compared with aged group. This experiment was replicated three times.

### Reactive oxygen species (ROS) detection

Intracellular levels of ROS were evaluated with 2′,7′-dichlorofluorescin diacetate (H_2_DCFDA). This molecule is de-esterified and is converted into a highly fluorescent dye upon oxidation. Oocytes were incubated with 10 μM of H_2_DCFDA diluted in TCM199 during 25 min at 38.5 °C in an atmosphere of 5% CO_2_ and maximum humidity. Thereafter, oocytes were washed twice in PBS containing 4% BSA, transferred to a drop on a glass slide and imaged immediately under fluorescence using an epifluorescence microscope. The green fluorescence was observed using 470/40 (excitation) and 525/50 (emission) filter. The images were captured by the image software AxioVision™ and the MFI was quantified in individual oocytes by ImageJ™ software, set as 1.0 in the young group and compared to the MFI of the aged group. Young and aged groups were compared using *t*-test, as well as aged-Res and aged-Ph groups, that were compared with aged group. This experiment was replicated 4 times.

### Immunoblotting

After IVM, pools of 20 denuded oocytes were lysed in RIPA lysis buffer, supplemented with phenylmethanesulfonyl fluoride (PMSF), 2 × Halt phosphatase inhibitor cocktail (Thermo Fisher Scientific), and CLAP protease inhibitor cocktail. Total protein concentration was quantified using the Pierce™ BCA Protein Assay Kit (Thermo Fisher Scientific), according to the manufacturers’ instructions. Absorbance was measured in a BioTek Synergy HT multi-detection microplate reader (BioTek Instruments, Winooski, Vermont, United States).

Samples were denatured through dilution with Laemmli sample buffer (Bio-Rad, Hercules, CA, USA)/β-mercaptoethanol and heating at 95°C for 5 min. Then, protein samples (20 μg) were loaded into 8% Acrylamide TrisHCL gels (Bio-Rad) and separated by electrophoresis. The samples were transferred to Immuno-Blot™ PVDF membranes (Bio-Rad), which were then blocked for 1 h at room temperature, with TBS-T (137 mM NaCl, 19 mM Tris-base, 0.1% Tween-20, pH = 7.6) containing 5% non-fat dry milk. Finally, membranes were incubated overnight at 4 °C, at a 1:1000 dilution, with the following primary antibodies: anti-SIRT1 (9475S, Cell Signaling, Danvers, Massachusetts, United States), anti-SIRT2 (19,655–1-AP; ProteinTech, Manchester, United Kingdom), anti-SIRT3 (ab189860; Abcam, Manchester, United Kingdom), and anti-β-ACTIN (A2228). The next day, membranes were washed with TBS-T and incubated at room temperature for 1 h, with the corresponding HRP-conjugated secondary antibody, goat anti-rabbit (1,706,515; Bio-Rad), or goat anti-mouse (1,706,516; Bio-Rad). Clarity Western ECL Substrate (Bio-Rad) was used for protein detection, carried out in the ImageQuant LAS 500 system (GE Healthcare, Uppsala, Sweden). Protein band densities were quantified with the ImageJ™ software, and the results were normalized for the respective β-actin bands. Young and aged groups were compared using t-test, as well as aged-Res and aged-Ph groups, that were compared with aged group. This experiment was replicated three times.

### In vitro fertilization (IVF) and embryo development

Frozen semen from one bull with previously proven in vitro and in vivo fertility was used throughout the IVF experiment. The semen was thawed at 37 °C for 20 s and layered below sperm Tyrode’s albumin, lactate and pyruvate medium (TALP) supplemented with 0.8 mg/mL pyruvic acid (P3662) and 0.1 mg/mL gentamycin (G1522) and incubated 1 h at 39 °C in a 5% CO_2_ in humidified atmosphere to allow recovery of motile spermatozoa (SPZ) through the swim-up procedure [67].

After incubation, the upper two thirds of the medium were recovered and centrifuged at 200 × *g* for 10 min. The sperm pellet was re-suspended in fertilization TALP medium supplemented with 0.03 mg/mL heparin, PHE (Lactate-Bisulfate solution with 0.169 mg/mL penicilliamine, 6.19 mg/mL hypotaurine, and 0.412 μg/mL epinephrine), 0.022 mg/mL pyruvic acid and gentamycin for in vitro insemination.

COCs were fertilized with 1 × 10^6^ SPZ/mL by co-incubation for 48 h at 5% CO_2_ in humidified atmosphere at 39 °C. Afterward, cumulus cells were removed by vortexing for 3 min and careful pipetting. In vitro culture of embryos was performed in synthetic oviduct fluid (SOF) medium supplemented with 2% essential amino acid solution (B6766), 1% non-essential amino acid solution (M7145), ITS (insulin 5 μg/mL, transferrin 5 μg/mL and selenium 5 ng/mL; I3146), and 0.1 mg/mL myo-inositol (I7508) in four-well plates under mineral oil at 5% O_2_ and 5% CO_2_ in humidified atmosphere at 39 °C. Fertilization and embryo development rates were evaluated at day 2 and day 7 (day 0 = insemination), respectively. Groups were compared using the chi-squared test. When assumptions were not met, data were analyzed in 2 × 2 contingency tables using Fisher’s exact test. This experiment was replicated 4 times and at least 20 oocytes per group were employed in each replicate.

### Statistical analysis

Statistical analysis was done using the IBM SPSS Statistics software (version 29, New York, USA). Data are presented as percentage or mean ± SEM. The level of statistical significance was set at *α* = 0.05. Shapiro–Wilk’s test was used to verify the distribution of variables and Levene’s test was used to verify equality of variances. To determine significant differences between groups (young versus aged and aged versus aged-Res or aged-Ph) continuous variables were evaluated using *t-*test. Two-sided *P* values were indicated. To verify if the mean SIRT1 levels were higher in the treatment groups (aged-Res or aged-Ph) versus control group (aged), one-sided *P* values were indicated, since it is well demonstrated that resveratrol is a SIRT1 activator in both somatic [45] and reproductive cells [68]. Categorical variables were analyzed using the chi-squared test. When assumptions were not met, data were analyzed in 2 × 2 contingency tables using Fisher’s exact test.

## Results

### Resveratrol and phloretin rescued the functional status of mitochondria in aged oocytes

**Fig. 1:**
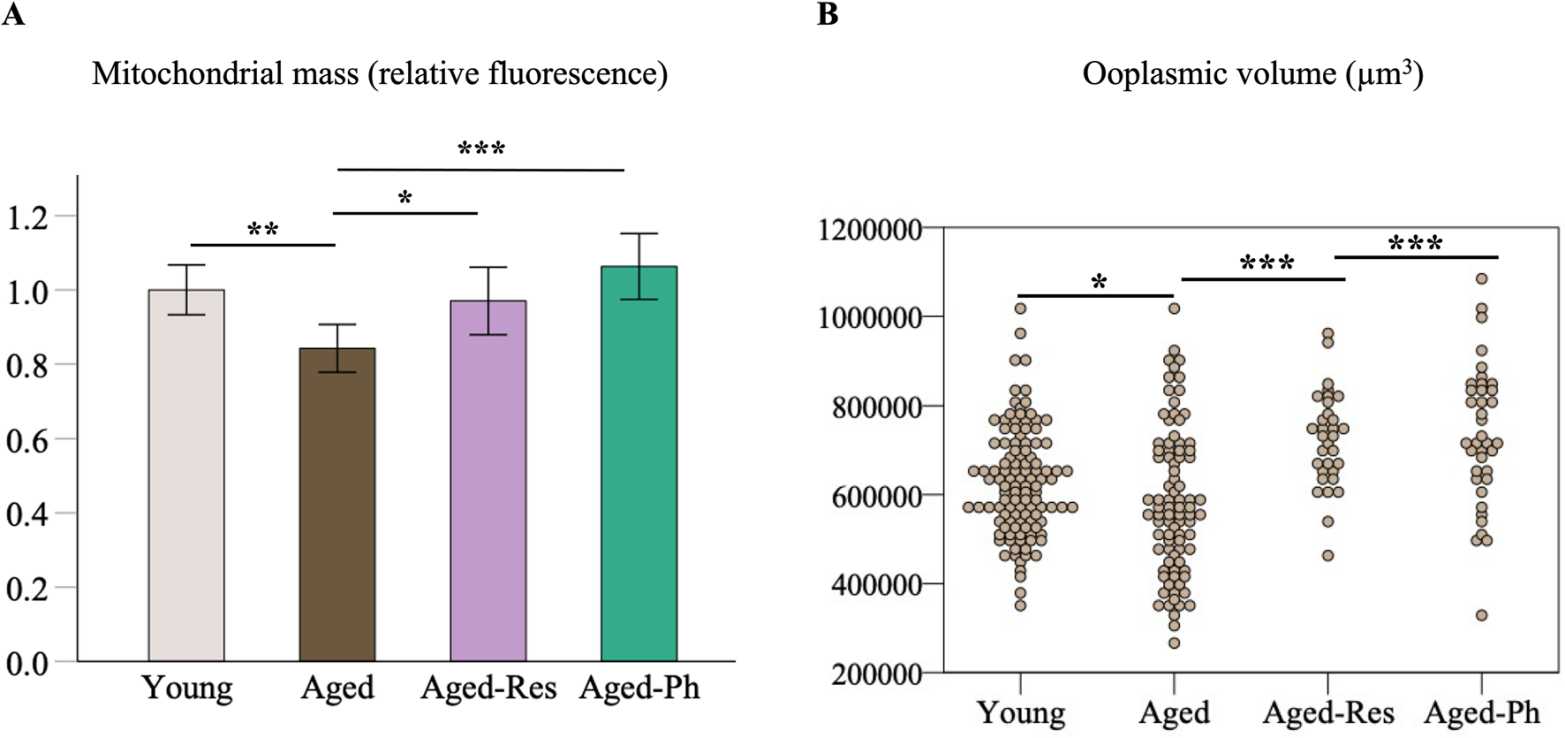
Comparison of the mitochondrial mass and ooplasmic volume. **A** The relative mass of active mitochondria was significantly lower in aged oocytes (*n* = 44) in comparison to young oocytes (*n* = 70) and was restored in the aged-Res (*n* = 25) and aged-Ph (*n* = 27). Bars represent the mean ± SEM. **B** Dots represent the volume of each oocyte measured in the IVM groups. The mean ooplasmic volume of aged oocytes (*n* = 87) was lower than the mean volume of young oocytes (*n* = 112) and was restored in oocytes matured with supplemented IVM medium: aged-Res (*n* = 32) and aged-Ph (*n* = 38). SEM: standard error of the mean. *P < 0.05, **P < 0.01, ***P < 0.001

The mean relative mass of active mitochondria was significantly lower in aged oocytes (0.842 ± 0.032, *P* = 0.002) in comparison to young oocytes (1.0 ± 0.034). The Aged-Res (0.970 ± 0.045, *P* = 0.023) and Aged-Ph (1.063 ± 0.044, *P* < 0.001) groups had a significantly higher mitochondrial mass in comparison to aged oocytes (Fig. 1A).

The mean ooplasmic volume (μm^3^) was lower in aged oocytes (585,674 ± 17,628) than in young oocytes (627,692 ± 11,463, *P* = 0.047) and was restored in both aged-Res (723,713 ± 18,865, *P* < 0.001) and aged-Ph (733,118 ± 25,456, *P* < 0.001) oocytes (Fig. 1B).

**Fig. 2:**
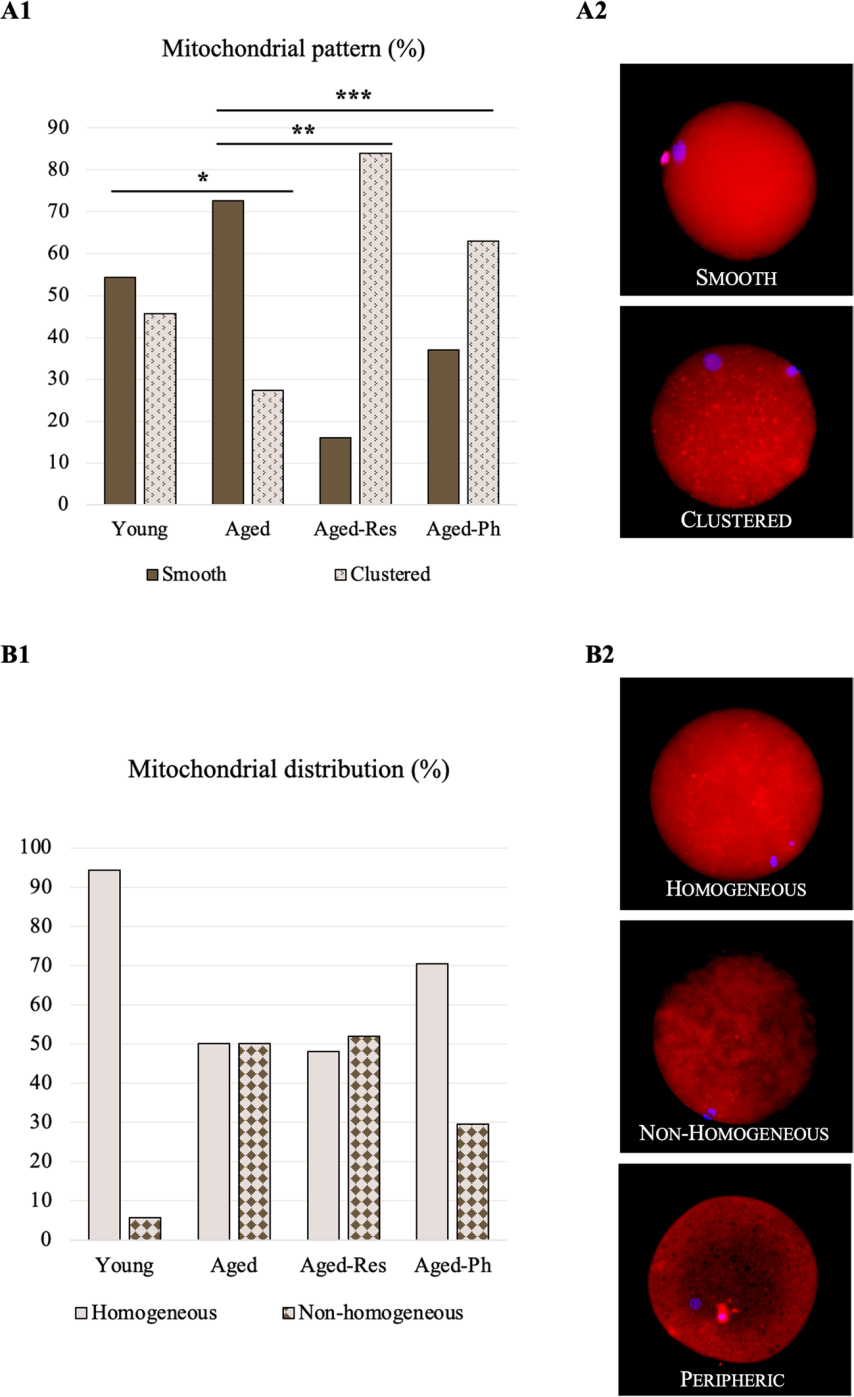
Comparison of the mitochondrial pattern and distribution. (**A1**) Aged oocytes had a lower proportion of oocytes with a clustered pattern when compared to young oocytes. This feature was recovered when IVM medium was supplemented with resveratrol or phloretin. Bars represent the percentage of each pattern in the IVM group. *(**A2**) Representative images of smooth and clustered patterns of oocytes stained with MitoTracker™ Red CMXRos. Magnification: X 400. (**B1**) Aged oocytes had a lower proportion of homogenous mitochondrial distribution when compared to young oocytes, but the percentage of homogeneous distribution was not recovered in the aged-Res and aged-Ph groups. Bars represent the percentage of homogeneous and non-homogeneous (including the non-homogenous and peripheric classification) in each IVM group. (**B2**) Representative images of homogeneous, non-homogenous, and peripheric mitochondrial distribution of oocytes stained with MitoTracker.™ Red CMXRos. Magnification: × 400. *P < 0.05, **P < 0.01, ***P < 0.001

Furthermore, aged oocytes had a lower proportion of oocytes with a clustered pattern when compared to young oocytes (27.3% versus 45.7%, *P* = 0.049). When aged oocytes were matured in supplemented IVM medium, there was a higher proportion of clustered oocytes, corresponding to 84.0% in the aged-Res (*P* < 0.001) and 63.0% in the aged-Ph (*P* = 0.006) groups (Fig. 2(A1)). Aged oocytes also had a higher proportion of non-homogenous mitochondrial distribution (including the non-homogenous and peripheric classification) when compared to young oocytes (50.0% versus 5.7%, *P* < 0.001), in which the homogenous distribution represented 94.3%. However, in this case, the proportion of oocytes with a homogeneous distribution in the aged-Res (48.0%, *P* = 1.000) and aged-Ph (70.4%, *P* = 0.137) groups were not significantly different from the aged group (Fig. 2(B1)).

Taken together these results suggest that resveratrol and phloretin may rescue the functional status of oocyte mitochondria, according to restored ooplasmic volume, mitochondrial mass, and mitochondrial pattern.

### Resveratrol and phloretin restored ROS levels in aged oocytes.

**Fig. 3:**
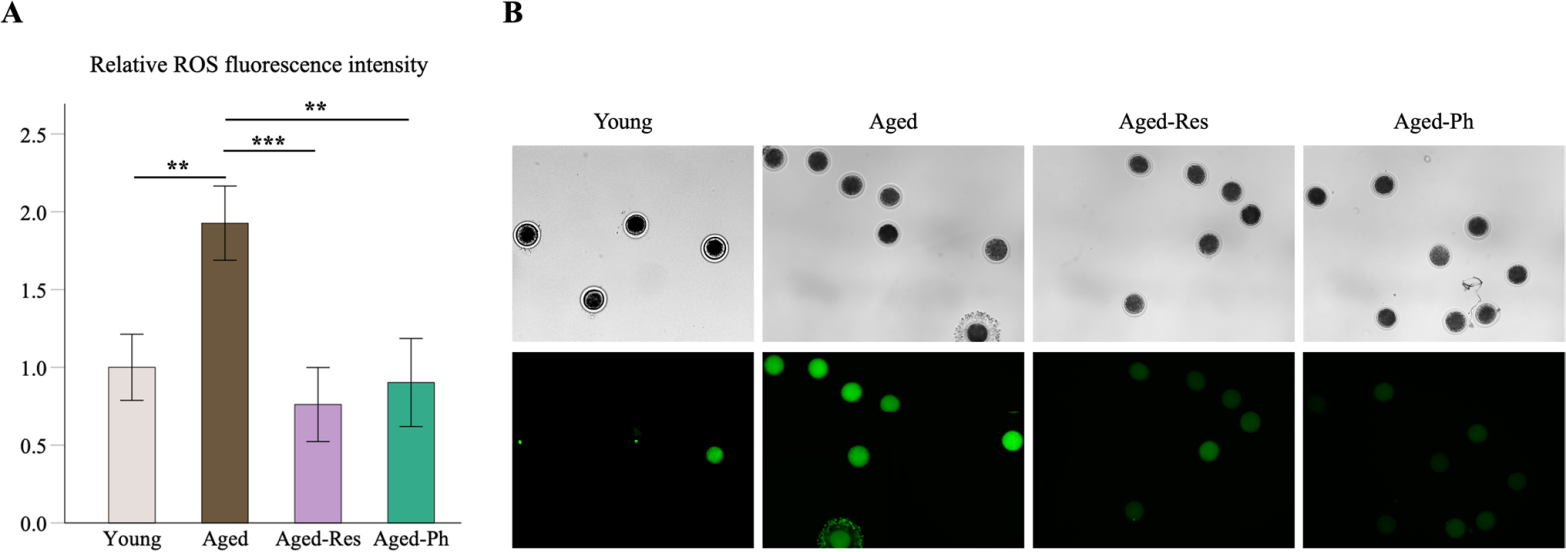
Comparison of relative ROS levels. **A** The relative fluorescence intensity of aged oocytes (*n* = 78) was 2.1 times higher than young oocytes (*n* = 81) and ROS levels were restored in the aged-Res (*n* = 43) and aged-Ph (*n* = 39) groups. Bars represent the mean ± SEM. **B** Representative images of ROS levels detected by dichlorofluorescin (DCF) fluorescence. Upper images were captured by phase contrast and below are the same oocytes captured by fluorescence microscopy at 100 × magnification. SEM: standard error of the mean. *P < 0.05, **P < 0.01, ***P < 0.001

The mean relative fluorescence intensity of aged oocytes (1.927 ± 0.239, *P* = 0.004) was two times higher than in young oocytes indicating an accumulation of ROS levels during postovulatory aging. Importantly, these levels were restored in the aged-Res (0.761 ± 0.238, *P* < 0.001) and aged-Ph (0.902 ± 0.283, *P* = 0.007) groups (Fig. 3), indicating that resveratrol/phloretin prevented the accumulation of ROS.

### Resveratrol restored the diminished levels of SIRT1 in aged oocytes, but postovulatory aging did not affect levels of SIRT2 and SIRT3

In our previous work, we have demonstrated an increase in the expression of SIRT1-3 during IVM, as well as the colocalization of those SIRTs with metaphase spindles, indicating that SIRT may have a role in meiotic spindle assembly [69]. Therefore, we sought to characterize the relative abundance of SIRT 1, 2, and 3 proteins in postovulatory aged oocytes with standard and supplemented IVM medium.

**Fig. 4:**
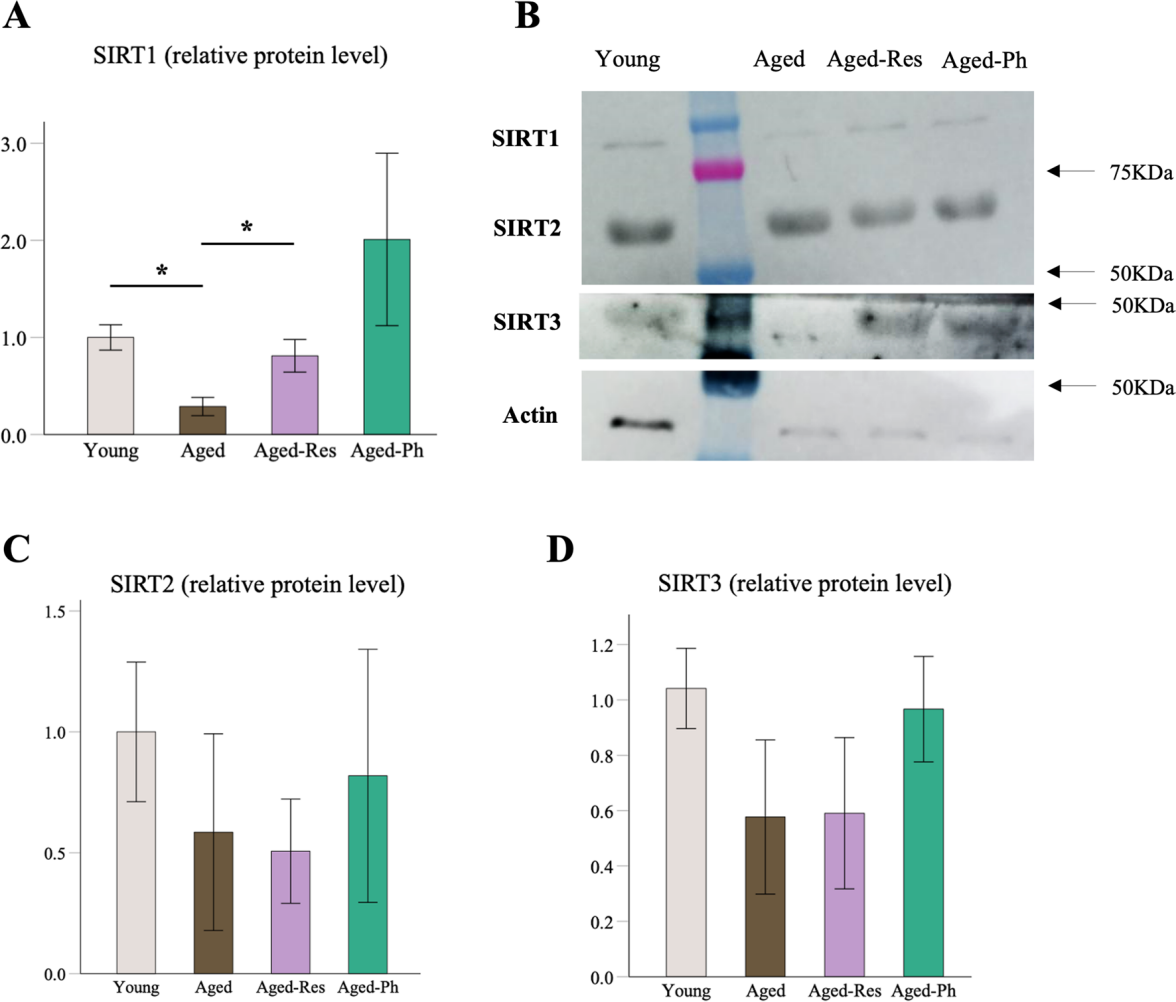
Comparison of SIRTs relative protein levels. The mean expression of SIRT protein level in the young group was set as 1.0. Bars represent the mean ± SEM. **A** The relative protein level of SIRT1 was 2.8 times higher in Aged-Res (0.811 ± 0.168, *P* = 0.027) in comparison to the aged group. The level of SIRT1 was not significantly different in aged-Ph versus aged (2.009 ± 0.888, *P* = 0.063). **B** Western blot analysis of SIRT1, SIRT2, and SIRT3. **C** The mean relative level of SIRT2 was 0.585, 0.507, and 0.818 in aged, aged-Res, and aged-Ph groups, respectively. **D** The mean relative level of SIRT3 was 0.577, 0.591, and 0.967 in aged, aged-Res, and aged-Ph groups, respectively. SEM, standard error of the mean. *P < 0.05, **P < 0.01, ***P < 0.001

We found lower mean SIRT1 relative protein levels in the aged group (0.289 ± 0.094, *P* = 0.004) in comparison to the mean level of the young group, set as 1.0. Moreover, the relative protein level of SIRT1 was 2.8 times higher in Aged-Res (0.811 ± 0.168, *P* = 0.027) in comparison to the aged group (Fig. 4A, B). The level of SIRT1 was not significantly higher in aged-Ph (2.009 ± 0.888, *P* = 0.063).

Levels of SIRT2 and SIRT3 were not significantly different between young versus aged groups nor aged versus aged-Res or aged-Ph groups (Fig. 4C, D).

### Embryo rates were decreased when aged oocytes were fertilized after IVM, though IVM supplementation with resveratrol or phloretin was insufficient to restore blastocyst developmental rate

Despite the similar cleavage rates between groups, aged oocytes had a lower developmental competence in comparison to young oocytes, presenting a lower blastocyst rate (1.4% versus 12.2%, *P* = 0.018), indicating that postovulatory oocytes have a lower quality, thus validating our experimental model.

**Fig. 5:**
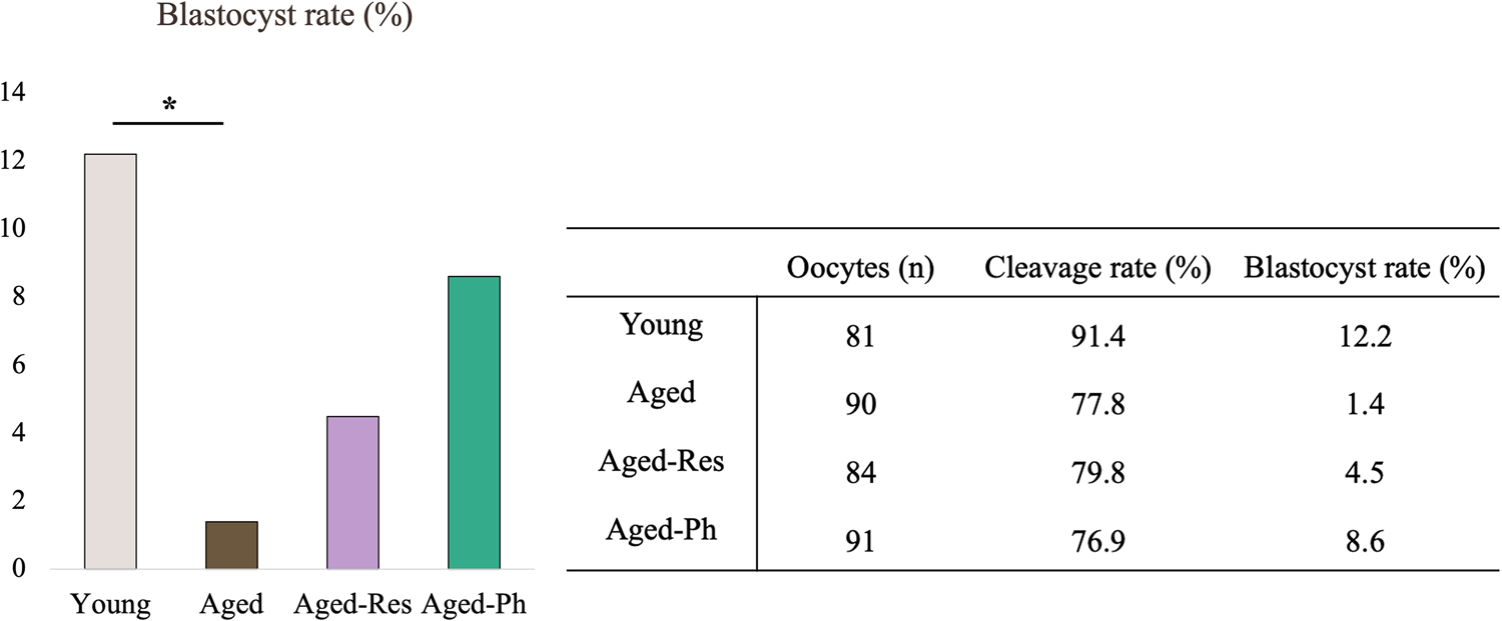
Comparison of embryo development. Bars represent the percentage of blastocysts in each group. The table indicates the number of oocytes in each group and the cleavage and blastocyst rates. *P < 0.05, **P < 0.01, ***P < 0.001

The blastocyst rate of aged-Res and aged-Ph was 4.5% (*P* = 0.359) and 8.6% (*P* = 0.116), respectively, which was not significantly different from the control group (aged oocytes), indicating that resveratrol/phloretin could not restore the oocyte competence of postovulatory aged oocytes (Fig. 5).

## Discussion

Our study demonstrated that the antioxidants resveratrol and phloretin can both revert hallmarks of age-related oxidative stress and mitochondrial dysfunction in bovine oocytes. Particularly, we documented for the first time the effect of in vitro supplementation of phloretin during in vitro maturation, corroborating its antioxidant effect previously documented in cells from other tissues and systems [61–63]. Nevertheless, embryo developmental rates were not significantly improved in the antioxidant groups, suggesting that age-related oocyte competence decline in postovulatory aging may not be limited to mitochondrial dysfunction.

Our findings also support the use of the bovine model of postovulatory aging to study age-associated phenomena and therapeutic interventions. Indeed, bovine oocyte and embryo development increasingly serves as a well-accepted model for studies on human early development [65].

Supplementation with resveratrol increased the active mitochondrial mass of aged bovine oocytes, a finding also reported in mice [51] and porcine species [53]. Several studies in which aged oocytes were supplemented with resveratrol reported restored levels of mitochondrial activity (higher mitochondrial membrane potential-MMP, and ATP levels) and content (mitochondrial mass and mtDNA) in association with higher maturation rates, lower spindle defects, and higher embryo developmental rate, highlighting the importance of mitochondria in oocyte competence [40, 51, 53, 54, 70]. In addition, aged oocytes supplemented with resveratrol/phloretin recovered the clustered pattern that characterizes young oocytes. Mitochondrial reorganization in clusters has been associated with higher mitochondrial activity and oocyte developmental competence [32, 71–73].

Age-related oxidative stress was noticeably reduced using resveratrol/phloretin. Comparable findings using resveratrol were obtained by Abbasi et al. in similar experimental conditions (i.e., in vitro aged oocytes supplemented with 2 μM of resveratrol) though using porcine oocytes [53]. Age-related oxidative stress has been demonstrated in oocytes from older females [18–21] and in vitro aged oocytes [74], in association with meiotic spindle defects [75–77] fertilization failure and decreased embryo development [19, 78–81]. Concomitant with an increase in ROS exposure, aged human, mice, and bovine oocytes seem to have a compromised antioxidant capacity (for review, see Ferreira et al. 2023).

The role of SIRTs in female reproductive cells is still being investigated. Even though SIRTs are not required for follicle development [82], increasing levels of SIRT1 [19] and SIRT3 [83] have been reported during oocyte maturation and inhibition of SIRT1 [29, 84] was shown to significantly decrease the rate of oocyte maturation, indicating that SIRTs levels may have a role during oocyte maturation and postfertilization events. In our previous work, we have demonstrated that SIRT1-3 are colocalized with metaphase spindles, suggesting that those SIRTs may have a role in the meiotic spindle assembly and microtubule dynamics [69]. Importantly, lower levels of SIRTs have been documented in oocytes from old females [21, 29, 30] and postovulatory aging [77, 78, 81, 85–89] in association with detrimental oocyte quality.

We encountered decreased levels of SIRT1 in aged oocytes, that were only restored by in vitro supplementation with resveratrol (not phloretin), as previously demonstrated in oocytes from aged cows [19, 54] and mice [40]. Systemic administration of resveratrol by short-term intraperitoneal injection resulted in higher SIRT1 [55] and a correlation between serum levels of resveratrol and ovarian levels of SIRTs has been reported in mouse after oral supplementation, also correlated with implantation and live offspring rates [70]. In our study, decreased oxidative stress by resveratrol was possibly mediated by SIRT1, which was significantly higher in aged-Res in comparison to aged (control) oocytes. Although phloretin regulates the Sirt-1/AMPK pathway in mouse hepatocytes [90], the similar level of SIRT1 between aged-Ph and aged oocytes in our study indicates that phloretin may exert its antioxidant effect by another mechanism [61, 62].

Since supplementation of IVM medium with antioxidants restored the active mitochondrial mass, ROS levels, and SIRT1 (resveratrol), we were anticipating a higher blastocyst rate in supplemented groups. Nevertheless, the developmental rates were not significantly different in the antioxidant groups in comparison to the control group.

In vitro studies using old cows also revealed a higher SIRT1 expression [19, 54] and higher mitochondrial activity and content [54], although with differing fertilization rates (increased in one study [19]) but without differences in the other [54]. Embryo development was reportedly higher in the resveratrol group (blastocyst rate of 6.3% versus 0.6%) in the study conducted by Sugiyama et al. in association with higher expression of genes related to high developmental competence in granulosa cells (GC). In vivo [40] and in vitro [51, 52] studies conducted in mouse have also reported higher fertilization and blastocyst rates with resveratrol treatment.

Therefore, our data suggests that age-related oocyte competence decline in postovulatory aging may not be limited to mitochondrial dysfunction and involve other organelles and fundamental processes, such as dysfunction of the endoplasmic reticulum, impaired calcium homeostasis, apoptosis, and degradation of proteins and transcripts, areas in which research is ongoing (for review, see Ferreira et al*.* 2023).

This was the first study to explore in vitro antioxidant properties of phloretin in the reproductive field. Indeed, phloretin was able to prevent ROS accumulation and preserve mitochondrial function in aged oocytes. While research assessing the role of antioxidants in improving female fertility is mostly from mouse studies, our study has the advantage of using a bovine model, which is considered a better model to study human reproductive physiology [91, 92].

Although the biological consequences of preovulatory (i.e., aged females) and postovulatory aging on oocytes largely overlap, resulting in spindle abnormalities and lower embryo developmental rates, the underlying mechanisms may be distinct, which represents a limitation of our study. Another limitation is the animal model since bovine and human reproductive cells may respond differently. On the other hand, studies using human oocytes rely on material obtained at the time of oocyte pick-up during ART treatments (from follicular puncture and aspiration), which may also represent a bias, because they are achieved from an infertile population. Hence, this should also be considered when interpreting results.

## Conclusions

The antioxidants resveratrol and phloretin can both revert the age-related oxidative stress and mitochondrial dysfunction during postovulatory aging but were insufficient to enhance embryo developmental rate under our experimental conditions. Systemic supplementation of aged females with those antioxidants should be further tested in pre-clinical and clinical studies, also addressing the implication of other organelles and fundamental processes in oocyte aging.

### Supplementary Information

Below is the link to the electronic supplementary material.

Supplementary file1 (DOCX 18 KB)

Supplementary file2 (DOCX 18 KB)

## Data Availability

The datasets used and/or analyzed during the current study are available from the corresponding author on reasonable request.
