# Supplementary material for: Chemical reversion of age-related oocyte dysfunction fails to enhance embryo development in a bovine model of postovulatory aging
Source: J Assist Reprod Genet. 2024 Jun 1;41(8):1997–2009. doi: 10.1007/s10815-024-03151-4 (PMC11339206; doi:10.1007/s10815-024-03151-4)
Supplement: Supplementary file 1 — Supplementary file1 (DOCX 18 KB) [file 10815_2024_3151_MOESM1_ESM.docx]

**Table 1. Supplementation of IVM medium using different concentrations of resveratrol**

| **IVM** | **Resveratrol** | **Maturation rate (%)** | **Morphological abnormal (%)** | **ROS-stained (%)** |
| --- | --- | --- | --- | --- |
| 23 h | --- | 80 | 0 | 17.6 |
|  | 2 μM | 57.1 | 0 | 0 |
|  | 20 μM | 70.5 | 16.7 | 0 |
|  | 100 μM | 53.8 | 35.7 | 50 |
| 30h | --- | 66.6 | 26.3 | 89.5 |
|  | 2 μM | 61.5 | 12.5 | 45.3 |
|  | 20 μM | 54.5 | 26.7 | 66.6 |
|  | 100 μM | 71.4 | 29.4 | 94.1 |
