# Supplementary material for: Chemical reversion of age-related oocyte dysfunction fails to enhance embryo development in a bovine model of postovulatory aging
Source: J Assist Reprod Genet. 2024 Jun 1;41(8):1997–2009. doi: 10.1007/s10815-024-03151-4 (PMC11339206; doi:10.1007/s10815-024-03151-4)
Supplement: Supplementary file 2 — Supplementary file2 (DOCX 18 KB) [file 10815_2024_3151_MOESM2_ESM.docx]

**Table 2. Supplementation of IVM medium using different concentrations of phloretin**

| **IVM** | **Phloretin** | **Maturation rate (%)** | **Morphological abnormal (%)** | **ROS-stained (%)** |
| --- | --- | --- | --- | --- |
| 23 h | --- | 79.2 | 4.0 | 32.0 |
|  | 2 μM | 66.7 | 0 | 75.0 |
|  | 6 μM | 62.5 | 0 | 37.0 |
|  | 10 μM | 70.0 | 4.0 | 45.8 |
|  | 50 μM | 35.3 | 16.7 | 91.7 |
|  | 200 μM | 22.2 | 11.1 | 72.2 |
| 30h | --- | 69.9 | 17.4 | 56.5 |
|  | 2 μM | 77.8 | 10 | 40.0 |
|  | 6 μM | 50.0 | 0 | 25.0 |
|  | 10 μM | 69.9 | 17.6 | 17.6 |
|  | 50 μM | 23.7 | 23.0 | 23.1 |
|  | 200 μM | 29.4 | 11.0 | 11 |
